# Supplementary material for: Effects of Trichoderma harzianum Strain T22 on the Arthropod Community Associated with Tomato Plants and on the Crop Performance in an Experimental Field
Source: Insects. 2022 Apr 28;13(5):418. doi: 10.3390/insects13050418 (PMC9147967; doi:10.3390/insects13050418)
Supplement: Supplementary file 1 [file insects-13-00418-s001.zip › insects-1687024-supplementary.pdf]

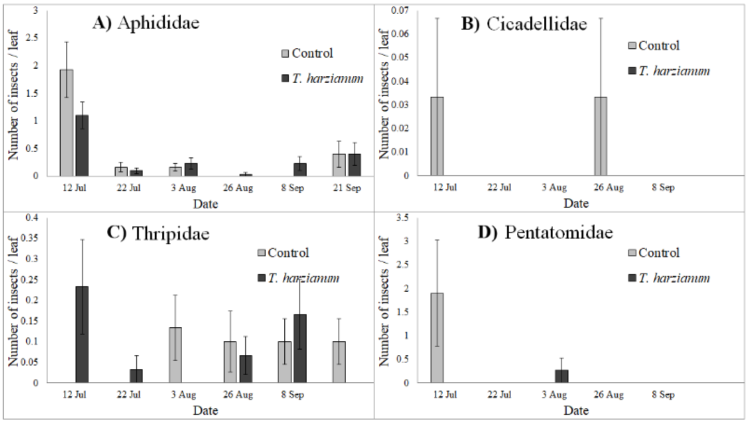

Figure S1. Families of piercing-sucking insects. Mean values (± standard errors) of insect abundance in the families of the piercing-sucking group on tomato leaves over time. At the family level, no significant differences in insect abundance were observed between treatments

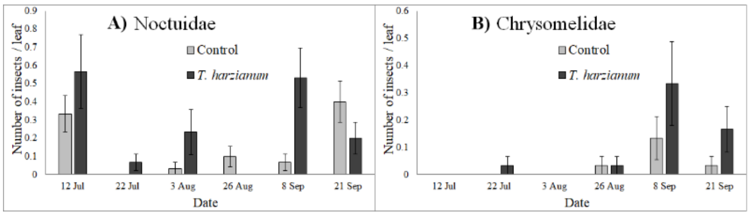

Figure S2. Families of chewing insects. Mean values (± standard errors) of insect abundance in the families of the chewing group on tomato leaves over time. At the family level, no significant differences in insect abundance were observed between treatments.

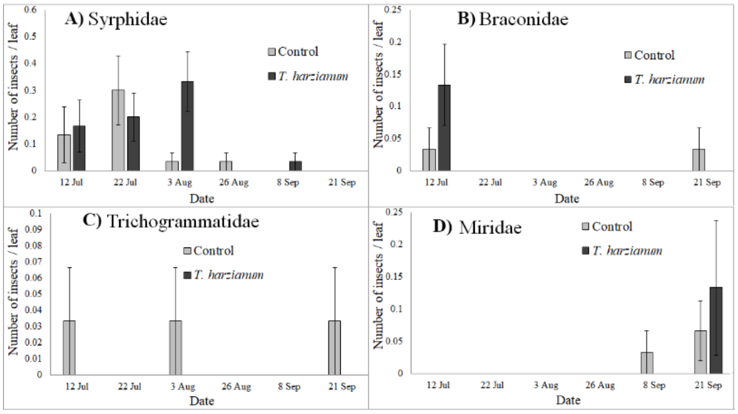

Figure S3. Families of natural enemies of insect. Mean values (± standard errors) of insect abundance in the families of natural enemies in tomato leaves over time. At the family level, no significant differences in insect abundance between treatments were observed
